# A New Encoding Architecture Based on Shift Multilayer Perceptron and Transformer for Medical Image Segmentation

**DOI:** 10.3390/s26020449

**Published:** 2026-01-09

**Authors:** Hepeng Zhong, Jieqiong Yang, Yingfei Wu, Jizheng Yi

**Affiliations:** 1 College of Advanced Interdisciplinary Studies, Central South University of Forestry and Technology, Changsha 410004, China; 20231100408@csuft.edu.cn (H.Z.); 20221200526@csuft.edu.cn (Y.W.); t20152279@csuft.edu.cn (J.Y.); 2 School of Mathematics and Physics, Hunan Institute of Technology, Hengyang 421002, China

**Keywords:** transformer, encoding architecture, shift multilayer perceptron, medical image

## Abstract

**Highlights:**

**What are the main findings?**

- A novel medical image segmentation framework integrating a Shift Multilayer Perceptron and a Transformer encoder is proposed, effectively capturing both low-level and long-range contextual dependencies.
- The incorporation of Senet Atrous Spatial Pyramid Pooling (SASPP) and the channel Feature Aggregation Attention Module (FAAM) enhances feature representation, achieving consistent improvements in Dice coefficients (87.01% on ACDC and 79.35% on Synapse) over state-of-the-art baselines.

**What are the implication of the main findings?**

- The proposed Multilayer Perceptron–Transformer (MPT) framework improves accuracy and generalization in multi-organ medical image segmentation, providing a robust foundation for clinical diagnosis and surgical planning.
- By optimizing feature fusion and mitigating information loss in U-shaped architectures, this work contributes to the evolution of Transformer–MLP hybrid models for efficient and precise medical image analysis.

**Abstract:**

Accurate medical image segmentation plays a crucial role in clinical diagnosis by precisely delineating diseased tissues and organs from various medical imaging modalities. However, existing segmentation methods often fail to effectively capture low-level structural details and exhibit inconsistencies in feature connection, which may compromise diagnostic reliability. To address these limitations, this study proposes a novel Multilayer Perceptron–Transformer encoding architecture that integrates the Shift Multilayer Perceptron and Transformer mechanisms. Specifically, a SENet-based Atrous Spatial Pyramid Pooling module is designed to extract multi-scale contextual representations, while the Shift MLP refines underlying spatial features. Moreover, a channel–feature aggregation attention module is introduced to strengthen information flow between the encoder and decoder layers. Experimental results on the Automatic Cardiac Diagnostic Challenge dataset show an average Dice Similarity Coefficient (DSC) of 87.01% (83.32% for the right ventricle, 90.90% for the left ventricle, and 86.83% for the myocardium). On the Synapse multi-organ segmentation dataset, the proposed model achieves an average DSC of 79.35% and a 95% Haus Dorff Distance of 20.07 mm. These results demonstrate that MPT effectively captures both local and global anatomical structures, providing reliable support for clinical diagnosis.

## 1. Introduction

Medical image segmentation plays a vital role in medical image analysis, enabling the accurate delineation of anatomical structures and pathological regions for diagnosis, treatment planning, and disease monitoring [1,2,3]. It provides clinicians with quantitative information that supports surgical navigation, radiotherapy planning, and the longitudinal follow-up of disease progression. However, medical image segmentation remains a challenging task due to the intrinsic complexity and variability of medical data. Images are acquired from diverse modalities such as Computed Tomography (CT), Magnetic Resonance Imaging (MRI), and ultrasound, each presenting distinct contrast mechanisms, noise characteristics, and spatial resolutions [4,5]. Moreover, substantial differences in organ morphology, boundary definition, and inter-patient variability further complicate segmentation tasks [6,7,8].

Before the deep learning era, medical image segmentation primarily relied on classical image processing techniques such as region growing, active contour models, and thresholding [9,10,11]. While these handcrafted methods could handle simple or high-contrast cases, they often failed in complex or noisy clinical environments, highlighting the need for adaptive models capable of learning semantic and structural cues directly from data. The advent of deep learning, particularly Convolutional Neural Networks (CNNs), fundamentally changed the paradigm of medical image segmentation by enabling automatic hierarchical feature extraction and end-to-end optimization [12]. These architectures effectively addressed the limitations of handcrafted methods that struggled with noise and structural variability. However, CNN-based models inherently emphasize local receptive fields, which restrict their ability to capture global contextual dependencies that are crucial for segmenting organs with complex geometries or low-contrast boundaries [13,14,15].

To mitigate this limitation, encoder–decoder architectures such as U-Net [16] introduced skip connections to recover spatial information lost during downsampling, significantly improving segmentation accuracy in small datasets with limited annotations. Despite its efficiency and widespread adoption [17,18,19,20], U-Net and its variants still face challenges in modeling long-range dependencies, maintaining structural continuity, and preserving fine-grained textures—particularly when dealing with multi-organ segmentation or images with blurred anatomical boundaries. These persistent challenges reveal a fundamental limitation of convolution-based architectures: their difficulty in jointly modeling fine-grained spatial continuity and global semantic context [21]. Consequently, recent studies have shifted toward Transformer-based designs to enhance global dependency modeling in medical image segmentation.

In pursuit of this balance, recent studies have introduced Transformer architectures [22], originally designed for natural language processing, to capture long-range dependencies in visual data. The self-attention mechanism in Transformers enables global feature interaction across all spatial locations, effectively compensating for the limited receptive field of CNNs and allowing the network to model holistic contextual relationships. In medical image segmentation, TransUNet [23] represents a pioneering hybrid design, integrating a CNN-based encoder with a Transformer module, achieving a comprehensive fusion of local spatial details and global semantic representations. Subsequent improvements, such as TransUNet++ [9] and Swin-UNet [24], refined the paradigm through hierarchical attention and shifted-window mechanisms, enhancing multi-scale feature aggregation and contextual reasoning. These Transformer-based frameworks have substantially improved segmentation accuracy and robustness across multiple benchmarks, establishing a new direction in globally contextual medical image segmentation [25,26,27,28,29,30].

However, the strong global reasoning capability of Transformers often comes at the expense of local texture and boundary precision, since self-attention prioritizes long-range interactions over spatial continuity. Moreover, empirical studies [31,32,33] have shown that repeated pooling and interpolation in U-shaped networks cause attenuation of boundary-level and texture-rich information, particularly in small or low-contrast structures. To address these challenges, we propose a Multilayer Perceptron–Transformer (MPT) framework embedded within a U-shaped encoder–decoder architecture to explicitly preserve low-level spatial features while maintaining global contextual understanding. The core of this framework is the Shift MLP, which performs spatial channel shifting before depthwise convolution, allowing each channel group to exchange information with neighboring positions and thus restore fine-grained spatial correlations weakened by pooling. By eliminating additional parameters, the design improves gradient stability and complements the Transformer’s global attention mechanism, ultimately achieving a balanced trade-off between local detail preservation and global reasoning.

To further improve multi-scale representation and feature fusion, a SENet-based Atrous Spatial Pyramid Pooling (SASPP) and a Feature Aggregation Attention Module (FAAM) are incorporated into the framework. The SASPP refines high-level semantic features across multiple receptive fields, while the FAAM enhances spatial–channel consistency, jointly improving robustness to complex anatomical structures and heterogeneous imaging conditions. The main contributions of this paper are as follows:

(1) A new Multilayer Perceptron Transformer framework is proposed to alleviate low-level feature degradation in U-shaped architectures, achieving a more balanced integration of local detail and global context through direction-aware channel shifting;
(2) A novel skip connection and FAAM-based fusion mechanism is introduced to improve cross-scale feature alignment and enhance the representation of complex spatial–channel relationships;
(3) Extensive experiments conducted on two benchmark datasets demonstrate that the proposed MPT framework outperforms several state-of-the-art segmentation methods in both accuracy and robustness.

## 2. Materials and Methods

**Figure 1:**
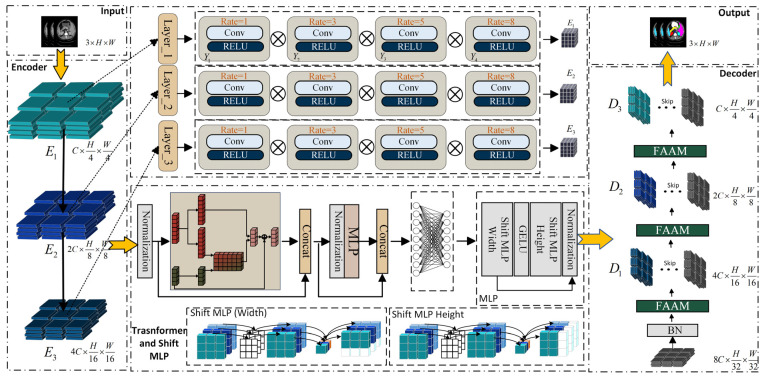
Overall architecture of the proposed Multilayer Perceptron–Transformer (MPT).

A new Multilayer Perceptron Transformer framework is proposed, as presented in Figure 1. It integrated the MLP mechanism into the encoding and decoding structure and enhanced medical image segmentation through an attention mechanism.

During the encoding process of the proposed network, multi-scale features are first extracted from the input image by adopting three Senet Atrous Spatial Pyramid Pooling (SASPP) modules. The original low-level features are preserved and reused in the decoder for cascaded feature fusion and upsampling. The multi-scale representations obtained through SASPP are then fed into the Transformer and Shift-MLP modules.

Then, the output of the multi-scale feature module is taken as the input to the Transformer. The self-attention mechanism of the Transformer is employed to enhance the long-range dependencies among image patches. Instead of explicitly adding positional encodings during patch embedding, a patch-based MLP is employed to implicitly encode positional information within the feature map after it passes through the Transformer module. Since the feature map already contains global information at this stage, it can effectively capture the position information through the convolutional layers. Subsequently, the image patches with the acquired position information will go through the MLP module to further highlight the underlying features of the encoding and decoding structure, and restore the features that integrate local and global semantic information, making their size match that of the feature map before being input into the Transformer. The decoding of the image involves a cascaded upsampling process. At each upsampling step, a channel attention enhancement module is incorporated to enhance the representational ability of features in the channel direction, and then linked with the original data in the previous encoding process. Finally, the cross-entropy loss function and the Dice loss function are used to perform gradient updates and backpropagation on the network to constrain the segmentation size of the image, so as to achieve the final segmentation task.

### 2.1. Senet Atrous Spatial Pyramid Pooling

To enhance the network’s feature extraction capability and overcome the difficulty of accurately segmenting organs with complex and overlapping voxels, a SENet-based Atrous Spatial Pyramid Pooling (SASPP) module is introduced, which leverages the multi-scale receptive fields of atrous convolutions and the channel reweighting mechanism of SENet to adaptively capture discriminative semantic features from medical images containing diverse spatial scales and intensity variations [34].

Firstly, the input of the encoding module $E_{i}$ is decomposed into sub-feature maps at multiple scales, represented as $Y_{1}$, $Y_{2}$……, $Y_{n}$. Features are extracted by using 3 × 3 convolutions with dilation rates of 1, 3, 5, and 8, followed by channel compression, ReLU activation, and feature restoration [35]. In the final step, the activation function sigmoid is applied to convert the channel values into a normalized range of 0-1. The resultant weight values are then multiplied by the original input features to yield the ultimate outcome of feature extraction. The formula of the algorithm is as follows:

(1) $$Y=C\mathrm{oncatenate}(\sum Relu(\mathrm{conv}(E_{i})),$$

Merge the result $Y\in R^{H/16\times W/16\times 4C}$ as an input parameter for the Transformer [36]. The algorithm of SASPP is shown in Algorithm 1.
**Algorithm 1:** Senet Atrous Spatial Pyramid PoolingInput: *encoder layer*$\;E_{i}$ $Step\;1:{\;Y}_{1}\leftarrow {Relu(conv}_{3\times 3}\left(E_{i},rate=1\right))$ $Step\;2:{\;Y}_{2}\leftarrow {Relu(conv}_{3\times 3}\left(E_{i},rate=3\right))$ $Step\;3:{\;Y}_{3}\leftarrow {Relu(conv}_{3\times 3}\left(E_{i},rate=5\right))$ $Input\;4:{\;Y}_{4}\leftarrow {Relu(conv}_{3\times 3}\left(E_{i},rate=8\right))$ $Input\;5:\;Y\leftarrow concatenate(Y_{1},Y_{2},Y_{3},Y_{4})$ **Output**: Y

### 2.2. Shift Multilayer Perceptron

After the SASPP module aggregates multi-scale feature representations, a Transformer module is employed to further model the global contextual dependencies among spatial regions [37]. The Transformer utilizes a self-attention mechanism to establish relationships between all feature tokens, enabling the network to capture long-range dependencies and construct a two-dimensional embedded feature sequence.

Formally, for the encoded feature map $X\in R^{L_{i}\times d_{i}}$, Weight matrix $W_{q}\in R^{d_{i}\times d_{k}}$, $W_{k}\in R^{d_{s}\times d_{k}}$, $W_{v}\in R^{d_{s}\times d_{i}}$, Calculate the query Q, key K, and value V in the first layer of feature attention as follows:

(2) $$Q=W_{q}X,$$

(3) $$K=W_{k}X,$$

(4) $$V=W_{v}X,$$

In the Transformer module, the output of the encoder serves as the input to the corresponding decoder layer.

(5) $$Q_{i}=QW_{i}^{Q},K_{i}=KW_{i}^{K},V_{i}=VW_{i}^{V},i=1,....12,$$

(6) $$head_{i}=\mathrm{Attention}(Q_{i},K_{i},V_{i})=\mathrm{Softmax}(\frac{Q_{i}K_{i}^{T}}{\sqrt{d_{k}}})V_{i},$$

(7) $$\mathrm{Multihead}\;(Q,K,V)=Concact(head_{1},...,head_{i}),$$

The input feature X is converted into a sequence of one-dimensional vectors through patch embedding. Each token is then projected by the corresponding weight matrices to obtain feature representations, after which an attention weight matrix is computed to capture the dependencies among features.

(8) $$head_{i}=\mathrm{Softmax}(\frac{Q_{i}K_{i}^{T}}{\sqrt{d_{k}}})V_{i},$$

By merging each $head_{i}$ into $Z_{i}=Concact(head_{1},...,head_{12})$ and performing dot-multiplication with weight $W_{o}$, the output result of the Transformer layer is obtained by:

(9) $$Z=Z_{i}W_{o},$$

To refine the feature activations produced by the Transformer encoder rather than actual mathematical eigenvalues, the Shift MLP reorganizes and reweights these activations along the spatial and channel dimensions, thereby enhancing discriminative regions and suppressing redundant responses. This refinement improves segmentation performance by strengthening boundary localization and preserving fine structural details, which are essential for accurate delineation of small and complex anatomical structures.

Before the shift operation, we permute the feature channels to promote cross-channel spatial interaction and avoid redundancy among grouped features; this enables channels from different semantic subspaces to exchange spatial context for more comprehensive feature learning. The proposed Shift MLP consists of two submodules $\left(MLP_{width},MLP_{height}\right)$. Inspired by the window-based attention in Swin Transformer, we apply horizontal shifts in $MLP_{width}$ followed by vertical shifts in $MLP_{height}$, enhancing local continuity while preserving the global dependencies established by the Transformer.

Firstly, the feature map X is transformed from $\left(B,H_{embed},W_{embed},E_{embed}\right)$ to $\left(B,E_{embed},H_{embed},W_{embed}\right)$ through an embedding operation. Secondly, after applying zero-padding with a padding size of 2, its dimension becomes $\left(B,E_{embed},H_{padded},W_{padded}\right)$. To balance computational efficiency and representational diversity, we empirically divide the feature map into five channel groups, each of size $\left(B,E_{embed/5},H_{padded},W_{padded}\right)$. Empirical tests show that using 4–6 groups yields stable segmentation performance, while excessive partitioning weakens receptive coverage and increases computational cost. The five-group configuration provides a good trade-off between local detail preservation and global feature diversity, consistent with similar channel-grouping strategies adopted in AS-MLP [38] and CycleMLP [39]. During the shift operation, zero values appear in the translated feature map, while the original valid features are shifted toward the edge regions that were initially zero-padded. Finally, through concatenation, each $X_{i}$ is recombined into a new feature map. Overall, it can be summarized as:

(10) $$X_{1}={\mathrm{Shift}}_{\mathrm{width}}(X),$$

(11) $$X_{2}=\mathrm{Embedding}(X_{1}),$$

(12) $$X_{3}=\mathrm{Gelu}\;(\mathrm{Dwconv}(X_{2})),$$

(13) $$X_{4}={\mathrm{Shift}}_{\mathrm{height}}(X_{3}),$$

(14) $$\mathrm{Output}(y)=Ln(X_{2}+\mathrm{Gelu}(\mathrm{Dwconv}(\mathrm{Embedding}(X_{5})))),$$

Each shift MLP block is composed of four layers and two shift modules, including a GELU activation and a normalization layer. After grouping, the features representation of the Shift MLP module is denoted as $\tau_{i}\in R^{w\times h\times c}$, where w, h, and c represent the width, height, and number of channels, respectively. The shift operation can be decomposed into two steps: (1) dividing the channels into several groups, and (2) shifting each group in different directions along the width and height dimensions.

The features are first shifted upward along the width dimension and then shifted leftward along the height dimension.

(15) τ1^1:w−1,:,:←τ10:w,:,:,τ2^1:w−1,:,:←τ20:w,:,:,τ3^1:w−1,:,:←τ30:w,:,:,τ4^1:w−1,:,:←τ40:w,:,:τ5^1:w−1,:,:←τ50:w,:,:τ1^:,0;h−1,:←τ1:,1;h,:,τ2^:,0;h−1,:←τ2:,1;h,:,τ3^:,0;h−1,:←τ3:,1;h,:,,τ4^:,0;h−1,:←τ4:,1;h,:,,τ5^:,0;h−1,:←τ5:,1;h,:,

After displacement, each patch absorbs visual content from adjacent patches, and communication between different spatial positions can be smoothly completed.

The GELU layer is defined as GELU(x)=xΦ(x), where Φ(x) Φ(x) is the standard Gaussian cumulative distribution function, defined as $\Phi (x)=\frac{1}{2}\left[1+erf(\frac{x}{\sqrt{2}})\right]$. The operation of the normalization layer is to calculate the mean $\mu =\frac{1}{c}\sum_{i=1}^{c}x_{i}$ and standard deviation $\sigma =\sqrt{\frac{1}{c}\sum_{i=1}^{c}{\left(x_{i}-\mu \right)}^{2}}$ from the given input vector $x=\left[x_{1},...,x_{c}\right]$, and standardize each term $\overset{¯}{x_{i}}=\frac{x_{i}-\mu }{\sigma },\forall i\in \left[1,c\right]$ in $x=\left[x_{1},...,x_{c}\right]$.

The utilization of depthwise separable convolution following another embedding operation and parameter passing serves two purposes: (1) It aids in encoding the positional information of MLP features. The convolutional layer within the MLP block effectively captures position information and outperforms standard position encoding techniques. Unlike the position encoding used in the Vision Transformer (ViT) [32], which requires interpolation when there is a resolution mismatch between training and testing, this approach avoids performance degradation. (2) Depthwise separable convolution also offers parameter efficiency, requiring significantly fewer parameters. The resulting features are then activated using GELU. Subsequently, the resulting features are then activated using the GELU function. Subsequently, the features undergo another MLP operation (across the height dimension) to further adjust the feature size. A residual connection is employed, incorporating the original token as the residual. Finally, layer normalization is applied to transfer the output features to the subsequent block.

### 2.3. Feature Aggregation Attention Module (FAAM)

Due to the high sensitivity of image features to positional information and the spatial semantic inconsistencies between two output feature maps, directly applying element-wise summation may lead to information redundancy or the loss of critical details [18]. To mitigate this issue, a Feature Aggregation Attention Module (FAAM) is introduced to adaptively align and fuse multi-scale features while preserving important spatial channel information.

**Figure 2:**
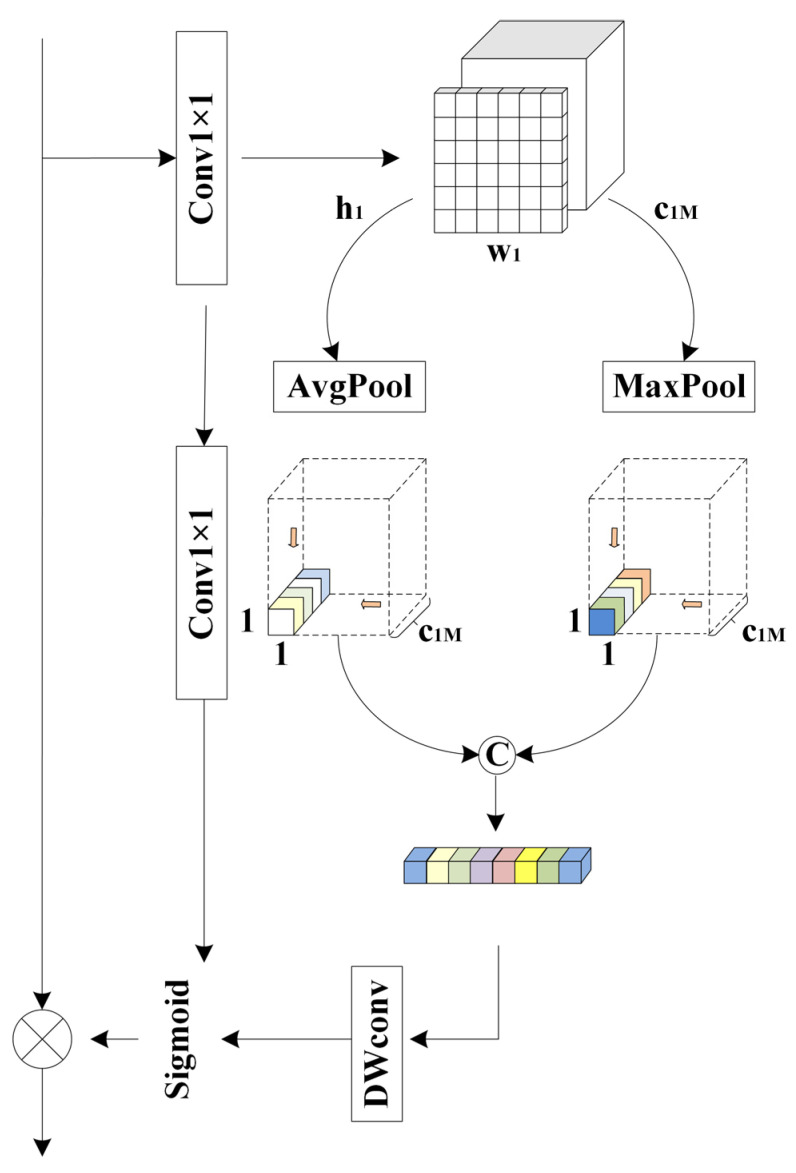
Feature aggregation attention module.

As shown in Figure 2, a new attention structure, FAAM, is proposed. To emphasize positional information, a new 1 × 1 convolutional layer is introduced to enhance the positional features, while another 1 × 1 convolutional layer is applied to compress the input feature map for semantic extraction, with a channel reduction ratio of 4. Then, average pooling and max pooling are used to compress the spatial dimension of the input features. A point convolution is then performed to aggregate spatial information and enhance the network’s representational capability. Semantic segmentation information is generated for two distinct spatial contexts, followed by a depthwise convolution (DW convolution) for further refinement. The detailed algorithm of FAAM is presented in Algorithm 2.
**Algorithm 2:** Feature Aggregation Attention ModuleInput: Block after connection X $\mathrm{Step}\;1\text{:}\;X_{t}\leftarrow X,X_{d}\leftarrow X,X_{c}\leftarrow {Conv}_{1\times 1}(X)$ $\mathrm{Step}\;2\text{:}\;X_{t1}\leftarrow Maxpool(X_{t}),X_{d1}\leftarrow Avgpool(X_{d})$ $\mathrm{Step}\;3\text{:}\;X_{t2}\leftarrow Point_{conv}(X_{t1}),X_{d2}\leftarrow Point_{conv}(X_{d1})$ $\mathrm{Step}\;4\text{:}\;X_{D}\leftarrow concatenate(X_{t2},X_{d2})$ $\mathrm{Step}\;5\text{:}\;Y\leftarrow concatenate(DWconv(X_{D}),X_{c})$ Output: New block Y

### 2.4. Spatial and Channel Skip

In U-shaped encoder–decoder architectures, the semantic gap between low-level encoder features and high-level decoder features often hinders accurate feature fusion. To alleviate this problem, a spatial and channel skip connection technique is employed to adaptively align and transmit complementary information between the encoder and decoder. Within this skip pathway, the feature maps are first divided along the channel dimension, and both max-pooling and average-pooling operations are applied to capture distinct activation responses. Unlike conventional skip connections that directly concatenate encoder features with decoder features, the proposed approach introduces an intermediate transformation through a sequence of residual convolutional layers, which refine and normalize the encoder features before fusion. This design ensures smoother feature transitions and reduces semantic inconsistencies between encoder and decoder stages. The algorithm of spatial and channel skip is shown in Algorithm 3.

**Figure 3:**
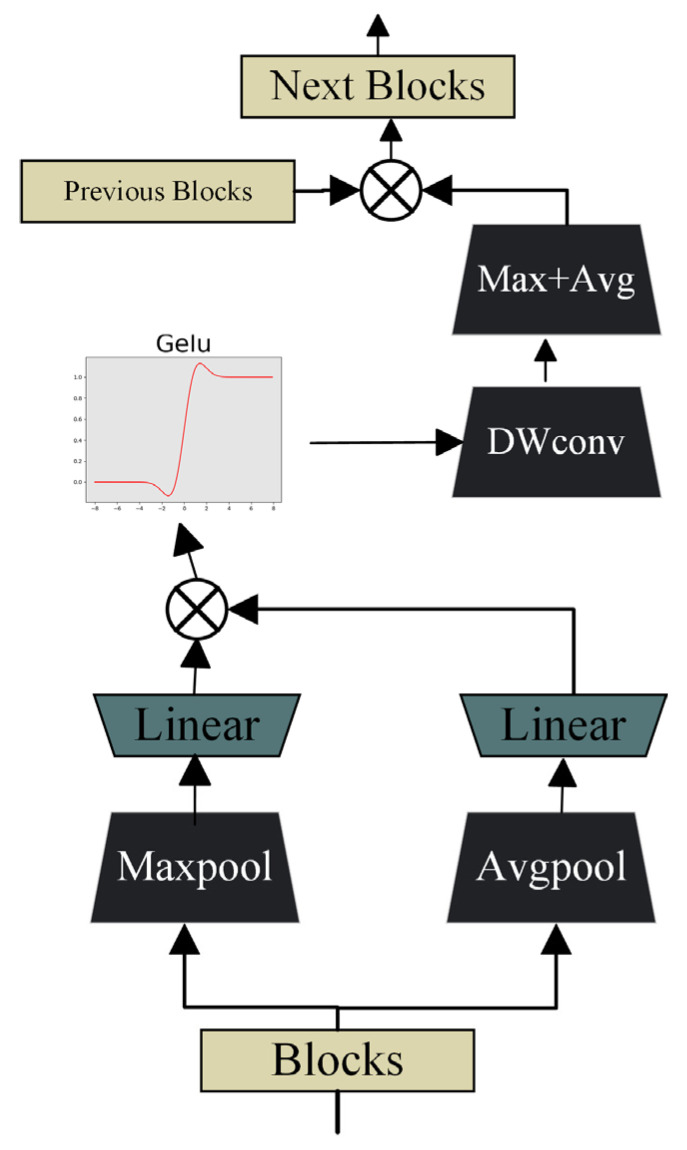
Design of the spatial and channel skip connection module.

As shown in Figure 3, for the $i_{th}$ block $D_{i}$ and its corresponding original image block $E_{i}$, ${MAX(D}_{i}$) and $AVG(D_{i}$*)* are utilized to enhance data features through a linear layer followed by activation functions. To further accelerate model convergence, Batch Normalization is incorporated after the linear layer to rescale the offset distribution of the data. This normalization ensures that the input values of the GELU activation function remain within its responsive range, thereby amplifying gradients, accelerating the learning process, and mitigating gradient vanishing issues.
**Algorithm 3:** Spatial and Channel Skip$\mathrm{Input}:\;Upsampled\;D_{i},Original\;E_{i}$ $Step1:X_{1}\leftarrow Maxpool\left(D_{i}\right),X_{2}\leftarrow Avgpool\left(D_{i}\right)$ $Step2{:X}_{3}\leftarrow Concatenate(Linear\left(X_{1}\right),Linear\left(X_{2}\right)$ $Step3{:X}_{4}\leftarrow DWConv(Gelu\left(X_{3}\right),kernels\left(1,1\right))$ $Step4{:X}_{5}\leftarrow Maxpool(Avgpool\left(X_{4}\right))$ $Step5:Y\leftarrow Concatenate(X_{5},E_{i})$ Output: Upsampled block Y

To perform local feature extraction, depthwise separable convolution is employed. Its primary objective is to precisely capture fine-grained spatial information within the feature maps. Compared with standard convolution, DWConv efficiently analyzes subtle differences and key features in each local region of the feature maps while maintaining a lower computational cost. After the DWConv operation, the resulting feature maps proceed to a stage of cascaded max pooling and average pooling, where the combination of pooling operations further enriches the feature set and enhances the network’s representational capacity.

Subsequently, the results are concatenated with $E_{i}$ to form the input of the FAAM in the next layer. The decoding stage can be mathematically formulated as:

(16) $$x_{i}=\mathrm{Encorder}(E_{i}),$$

(17) $$x_{t}=\sum \mathrm{FAAM}(D_{i}),$$

(18) $$D_{i+1}=\mathrm{skip}(x_{i},x_{t}),$$

## 3. Results

### 3.1. Data Introduction

As shown in Table 1, a total of 30 CT scans were selected, each consisting of 85–198 samples with a resolution of 512 × 512 and voxel sizes ranging from 0.54 × 0.98 × 2.5 mm^3^ to 0.54 × 0.98 × 5.0 mm^3^, resulting in 3779 axial contrast-enhanced abdominal CT images. All scans were obtained from the MICCAI 2015 Multi-Atlas Labeling Challenge dataset (Synapse). For eight abdominal organs (aorta, gallbladder, left kidney, right kidney, liver, pancreas, spleen, and stomach), the average Dice Similarity Coefficient (DSC) and average Hausdorff Distance (HD) were computed after randomly dividing the dataset into 18 training cases (2212 axial slices) and 12 validation cases. It should be noted that the Synapse dataset does not provide an official test set. Following the standard evaluation protocol adopted in previous works such as TransUNet [23], Swin-UNet [24], and MISSFormer [40], the 12 validation cases are used as the evaluation subset for reporting results. This is a commonly accepted practice to ensure fair comparison and reproducibility across the literature. To further verify the generalization capability of the proposed model, additional cross-dataset validation experiments were conducted between the Synapse (CT) and ACDC (MRI) datasets, which differ significantly in imaging modality, contrast, and anatomical structure.

Regarding the dataset utilized in the context of the Automatic Cardiac Diagnostic Challenge (ACDC), a sequence of short-axis slices derived from cine magnetic resonance imaging was employed. These slices were acquired during breath-holding and encompassed the heart, ranging from the base of the left ventricle to the apex, with a thickness ranging from 5 to 8mm. The spatial resolution in the short axis plane ranges from 0.83 to 1.75 mm^2^/voxel. The left ventricle (LV), right ventricle (RV), and myocardium (MYO) were manually annotated for each patient scan. A total of 70 cases (1930 slices) were used for model training, 10 cases (551 slices) for validation, and 20 cases (1102 slices) for testing, to compute the final average Dice Similarity Coefficient (DSC) score.

### 3.2. Evaluation Indicators

To effectively evaluate the performance of the proposed model, experiments were conducted on the Synapse multi-organ segmentation dataset and the Automatic Cardiac Diagnostic Challenge dataset. Two evaluation metrics, Dice Similarity Coefficient and 95% Hausdorff Distance, were used to assess segmentation performance. In addition, comparative analyses with other state-of-the-art models were performed to further validate the effectiveness of the proposed approach.

The DSC is a widely used set-based similarity metric for measuring the overlap between two samples, with values typically ranging from 0 to 1.

(19) $$DSC=\frac{2\left|A\cap B\right|}{\left|A\right|+\left|B\right|},$$

where A represents the predicted segmentation, and B denotes the ground truth segmentation. ∣A∩B∣ indicates the intersection between A and B, while ∣A∣ and ∣B∣ represent the number of elements in A and B, respectively. The coefficient 2 in the numerator accounts for the double-counting of shared elements between A and B in the denominator.

Hausdorff_95 is a metric that measures the degree of similarity between two sets of points. It is defined as a form of distance between two point sets. Assuming two sets, $A=\{a_{1},...,\;a_{p}\}$, $B=\{b_{1},...,\;b_{p}\}$, the calculation of HD95 between these two sets is given by Equation (20) and detailed as follows:

(1) Construct a distance matrix for A and B;
(2) Calculate the distance from each point a∈A to all points in B;
(3) Store these distances in a matrix D_A→B_;
(4) Similarly, for each point b∈B, calculate its distance to all points in A and store these distances in a matrix D_B→A_;
(5) Sort all values from the distance matrices D_A→B_ and D_B→A_ in ascending order;
(6) Find the 95th percentile value and denote it as HD95

(20) $$H\left(A,B\right)=max\left(max\left\{min||a-b||\right\},max\{min||b-a||\}\right),$$

where a∈A,b∈B, ||·|| denotes the L2 normal.

### 3.3. Implementation Details

All experiments were implemented in PyTorch 1.13.0 and conducted on a single NVIDIA RTX 3090 GPU (24 GB) with automatic mixed-precision training to reduce memory usage. Each input volume was normalized using z-score normalization on a per-volume basis, and the resulting 2D slices were resized to 512 × 512 pixels before being fed into the network. The model was optimized with stochastic gradient descent (learning rate = 0.005, momentum = 0.9, weight decay = 1 × 10^−4^). Online data augmentations—including random rotation (±15°), scaling (0.9–1.1), horizontal flipping, and intensity jittering (±10%)—were applied during training to enhance generalization. The batch size was 24, and the training ran for 20,000 and 14,000 iterations for the ACDC and Synapse datasets, respectively. During inference, each 3D volume was processed in a slice-wise manner, and the predicted 2D slices were stacked to reconstruct the 3D segmentation results for evaluation.

### 3.4. Loss Function

In the abdominal multi-organ segmentation task, significant structural differences among organs pose challenges to achieving high segmentation accuracy. To address this issue, a cross-entropy loss function with a boundary distance penalty term was adopted on the Synapse dataset. The corresponding formula is defined as follows:

(21) $$L_{CE}=-\sum_{i=1}^{n}y_{i}log(p_{i}),$$

(22) $$w(x,y)=exp(\frac{-d{\left(x,y\right)}^{2}}{\sigma^{2}}),$$

(23) $$L=L_{CE}+\lambda \sum_{x,y}w(w,y)\cdot M(x,y),$$

where $y_{i}$ denotes the ground-truth label, $p_{i}$ is the probability predicted by the model, d(x,y) represents the distance from voxel (x,y) to the nearest boundary voxel. The parameter σ controls the rate of weight decay. Voxels closer to the boundary are assigned higher weights, meaning segmentation errors on these voxels are penalized more severely. M(x,y) is an incorrect identifier at voxel (x,y). If voxel belongs to the organ region in the ground-truth label, then M(x,y)=1, otherwise, M(x,y)=0.

For the ACDC dataset segmentation task, a joint loss function was employed to optimize the training process, which can be formulated as

(24) $$L_{CL}=\alpha L_{CE}+\beta L_{Dice},$$

(25) $$L_{Dice}=1-\frac{2\sum_{i=1}^{n}p_{i}y_{i}}{\sum_{i=1}^{n}{y_{i}}^{2}+\sum_{i=1}^{n}{p_{i}}^{2}}$$

Thus, the category discrimination capability of the cross-entropy loss and the boundary sensitivity of the Dice loss are jointly exploited. This joint optimization enables the model to accurately segment vascular and myocardial tissues while effectively delineating structural boundaries, thereby enhancing the overall segmentation accuracy.

### 3.5. Experimental Results

#### 3.5.1. Comparative Experiments Analysis

As demonstrated in Table 2, it is evident that the proposed network achieves significant improvements in both average DSC and HD95, surpassing the V-net by 10.54% and showcasing a superior decoding strategy compared to direct upsampling. Similarly, when compared to the U-net, the hybrid encoder demonstrates a noteworthy 9.58% increase in DSC. Furthermore, in comparison to the TransUNet and Swin-UNet [24], the incorporation of skip connections in the proposed network leads to consistently satisfactory performance across various Transformer-based architectures. In addition, DA-TransUNet, VM-UNet, and CTC-Net demonstrate their respective strengths by leveraging dual-attention mechanisms, Mamba-based state space modeling, and convolution-enhanced transformer designs. In contrast, our method is built upon a distinct Shift MLP–Transformer hybrid framework and further enhanced by multi-scale context modeling and channel-wise feature aggregation. Although it does not achieve the absolute best performance on every metric, it consistently maintains competitive results across most evaluation criteria, indicating that it serves as a complementary solution to existing segmentation frameworks.

However, as depicted in Table 3, performance optimization remains unsatisfactory for the segmentation of the spleen and stomach. This limitation may arise from the MLP module, which primarily focuses on capturing high-level semantic representations, potentially at the expense of detailed texture information.

Additionally, the outcomes for the aorta and gallbladder still fall short compared to those achieved by conventional U-net networks. This could be attributed to the novel technological state introduced by the proposed Spatial and Channel skip connection when combined with the U-Net structure through skip connections.

Nevertheless, the network demonstrates a remarkable ability to learn high-level semantic features while preserving fine-grained structural details, which is particularly valuable in medical image segmentation. This observation is further supported by the consistent trend observed in the average HD95 metric.

**Figure 4:**
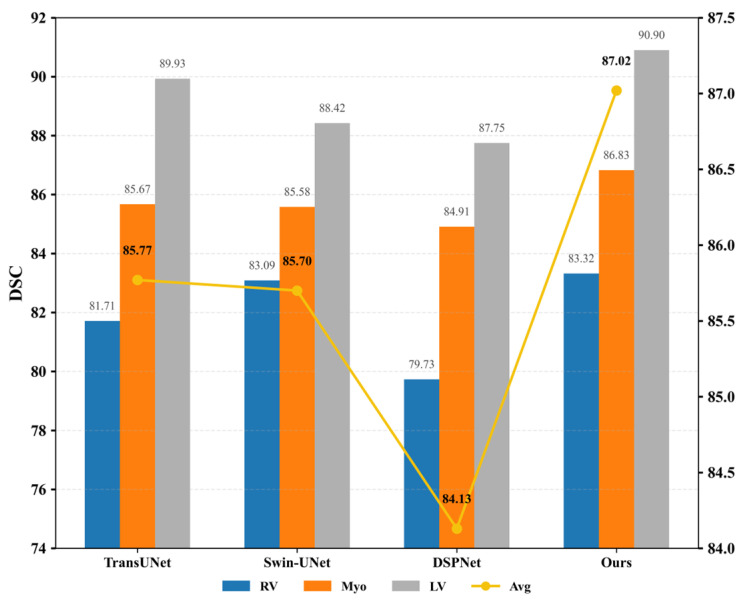
DSC values (left *Y*-axis, %) and HD95 scores (right *Y*-axis, mm) among TransUNet, Swin-UNet, DSPNet, and the proposed MPT on the ACDC dataset. The bar chart represents the Dice Similarity Coefficient (DSC, %), while the yellow line corresponds to the Hausdorff Distance (HD95, mm). Higher DSC and lower HD95 indicate better segmentation performance.

Secondly, as illustrated in Figure 4, the proposed model was compared with TransUNet and Swin-UNet on the ACDC dataset. The observed improvement can be attributed to the introduction of a novel pyramid-based atrous convolution pooling mechanism, which enhances spatial feature extraction. In the downsampling stage, spatial attention is strengthened to improve the capture of high-level semantic information, while in the upsampling stage, channel-wise attention is reinforced to refine feature restoration. This joint enhancement effectively benefits both high-level semantic learning and low-level detail detection.

#### 3.5.2. Ablation Experiment Analysis

To investigate the influence of different components on model performance, ablation experiments were conducted on the Synapse dataset.

**Figure 5:**
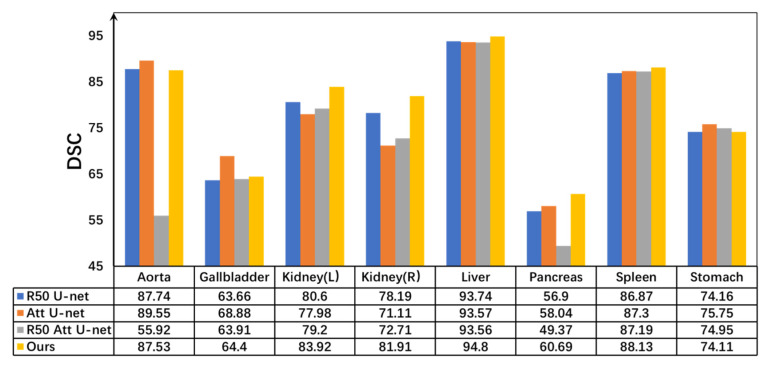
Dice comparisons of different U-shaped architectures and the proposed MPT on the Synapse dataset. All quantitative values are expressed as percentages (%). Higher values indicate better segmentation performance.

The proposed improvement strategies were compared with various U-Net-based architectures. As illustrated in Figure 5, the proposed network achieved notable gains in both average DSC and HD95 compared with traditional networks incorporating attention mechanisms. Furthermore, in the multi-class DSC metrics, the proposed model demonstrated outstanding segmentation performance, particularly for organs such as the kidney, liver, pancreas, and spleen. These results clearly indicate that the MLP module effectively captures high-level semantic information, thereby contributing to enhanced segmentation accuracy.

**Figure 6:**
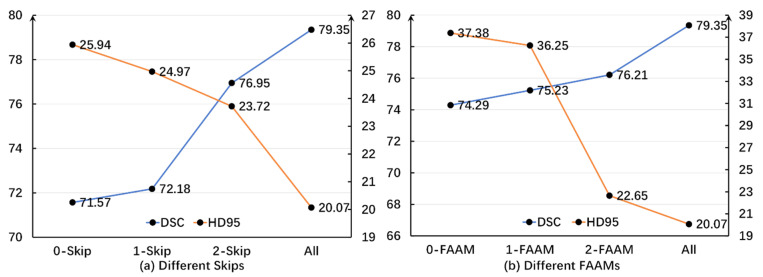
Distribution curves of DSC and HD95 under different numbers of skip connections and FAAMs on the Synapse dataset. DSC values are expressed as percentages (%), while HD95 is measured in millimeters (mm). A higher DSC and lower HD95 denote better segmentation accuracy.

To further validate the effectiveness of the skip connections and FAAMs, experiments were conducted using different numbers of skip connections and FAAMs, as illustrated in Figure 6.

In the various skip connections, it is evident that the HD95 value exhibits a consistent downward trend as the number of skips increases. This indicates a decrease in the error magnitude when compared to actual images. Additionally, there is a negative correlation between the number of skips and the accuracy of the algorithm. Similarly, regarding the DSC, although the initial introduction of skips did not lead to significant improvements, the trend related to skips becomes increasingly obvious with an increase or decrease in the number of skips. This demonstrates that the algorithm progressively enhances its similarity to the ground-truth segmentation, confirming the effectiveness of the skip connection strategy.

To further validate the efficacy of the FAAM, additional ablation experiments were conducted under a single-branch configuration. Similarly to the skip connection analysis, a negative correlation was observed between the number of FAAMs and HD95, while a positive correlation was found between the number of FAAMs and DSC. These results indicate that increasing the number of FAAMs progressively amplifies the inherent advantages of the proposed algorithm.

As presented in Table 4, it is evident that the SASPP technique demonstrates a robust recognition effect on the Synapse dataset, particularly for the segmentation of the aorta, pancreas, and spleen. Although the individual performance improvement of the MLP module is not significant, its combination with FAAM and MLP effectively showcases the segmentation efficacy for organs with subtle voxel differences during the pre-feature extraction and processing using MLP.

The FAAM has demonstrated significant improvements in recognizing the pancreas and spleen, especially considering the relatively small anatomical area of the pancreatic region in the whole human abdominal CT scan. Leveraging the specific characteristics of the pancreatic, FAAM, and SASPP utilize convolutional neural networks to achieve precise pancreatic segmentation.

To enable the segmentation network focus on extracting target regions while minimizing the inclusion of features from other regions, a strategy is adopted where the deeper layers of the network extract increasingly intricate features. Through multiple downsampling operations, complex and redundant information is successively discarded, leading to more accurate extraction of target region features. However, excessive downsampling can result in the loss of shallow spatial information, such as edges. While this improves the positioning accuracy of the pancreatic region, it can lead to a substantial loss of edge information in the final predicted image.

As shown in Table 5, on the ACDC dataset, the morphology of each cardiac region varies, leading to different segmentation methods and difficulties for each region. Crudely speaking, the left ventricle is a thick-walled cylindrical area, while the right ventricle is an irregularly shaped object, with thinner ventricular walls that are sometimes mixed with surrounding tissues.

Individually, every single module in the proposed framework yielded only marginal improvements in cardiac segmentation. However, when these modules were integrated, notable performance enhancements were achieved. In particular, the combination of the MLP, skip, and SASPP modules led to a significant improvement in the segmentation of the right ventricle (RV). This enhancement can be attributed to the multi-layer SASPP modules, which amplify the discriminative features of the RV, MYO, and LV, thereby increasing the contrast between the target and redundant regions. Subsequently, the Transformer-based processing enables more precise delineation of the target areas, ultimately resulting in higher segmentation accuracy in the final predictions.

#### 3.5.3. Statistical Significance Analysis

Finally, a statistical significance analysis was conducted to further assess the reliability of the proposed algorithm. For both the ACDC and Synapse datasets, independent samples *t*-tests were performed to evaluate the differences in segmentation performance between the proposed MPT framework and the comparative baseline methods. The *t*-tests were applied to determine whether the observed performance differences were statistically significant under a predefined confidence level, thereby providing a quantitative validation of the robustness and consistency of the proposed approach.

As shown in Table 6, the statistical significance analysis was conducted to validate the reliability and robustness of the proposed MPT framework on both the ACDC and Synapse datasets. Independent samples *t*-tests were applied to compare the segmentation performance of the proposed method with that of several representative baselines.

For the Synapse dataset, the MPT framework achieved higher mean Dice scores than all comparative methods. Among them, the improvement over V-Net reached statistical significance (t = 3.01, *p* = 0.016), indicating a clear performance advantage. Although the differences compared with DARR, TransUNet, and DSPNet did not reach the 0.05 significance level, they still demonstrated consistent positive mean gains, suggesting stable performance improvements.

For the ACDC dataset, MPT also outperformed all baseline models. The difference relative to U-Net was statistically significant (t = 2.87, *p* = 0.020), while the comparisons with Attention-UNet, Swin-UNet, and TransUNet exhibited smaller but consistent gains. These results confirm that the proposed architecture yields reliable and repeatable improvements across both MRI and CT segmentation tasks.

Overall, the *t*-test results support that the proposed MPT framework achieves statistically and practically meaningful performance advantages over conventional U-shaped architectures, thereby demonstrating enhanced robustness and generalization capability across heterogeneous medical imaging modalities.

#### 3.5.4. Experimental Efficiency Analysis

As shown in Table 7, the proposed MPT framework demonstrates clear advantages in computational efficiency compared with TransUNet and Swin-UNet. Specifically, MPT reduces parameters and FLOPs by approximately 19% and 35%, respectively, relative to Swin-UNet, while achieving 15% faster inference and 1.1 GB lower VRAM usage under the same input resolution on an RTX 3090 GPU. These improvements mainly result from the parameter-free Shift-MLP, the lightweight SASPP with SENet re-weighting, and the FAAM that reuses intermediate features to minimize redundant computation. Overall, MPT achieves a superior balance between segmentation accuracy and computational cost, enabling efficient and scalable medical image segmentation.

#### 3.5.5. Robustness Analysis Under Imaging Perturbations

To further assess the robustness of the proposed MPT framework under realistic imaging variations, additional experiments were conducted to simulate common perturbations that may occur in clinical acquisition scenarios. Two types of distortions were considered: Gaussian noise perturbation and voxel spacing distortion.

During inference, Gaussian noise with standard deviations of σ = 0.01, 0.03, and 0.05 was added to the input CT slices to simulate scanner-induced noise. In addition, spacing perturbations of ±5% and ±10% were applied to the axial voxel resolution to mimic spatial resampling or calibration inconsistencies during scanning. The quantitative results are summarized in Table 8.

As shown in the table, the MPT framework exhibits a moderate but consistent degradation trend with increasing perturbation levels, where the Dice score variation remains within 1.5%, and the HD95 fluctuation is less than 1.5 mm. This demonstrates that the proposed Feature Aggregation Attention Module and spatial–channel skip connections effectively enhance feature consistency and suppress sensitivity to noise and geometric distortions.

These findings confirm the robustness and stability of the proposed MPT architecture against common degradation factors in medical imaging. Furthermore, while large-scale cross-dataset experiments on 3D volumetric data are reserved for future work due to architectural and computational limitations, the current results on Synapse (CT) datasets, together with the above perturbation analyses, clearly demonstrate the strong cross-modality generalization and stability of the proposed MPT framework.

### 3.6. Visualization of Image Segmentation Results

**Figure 7:**
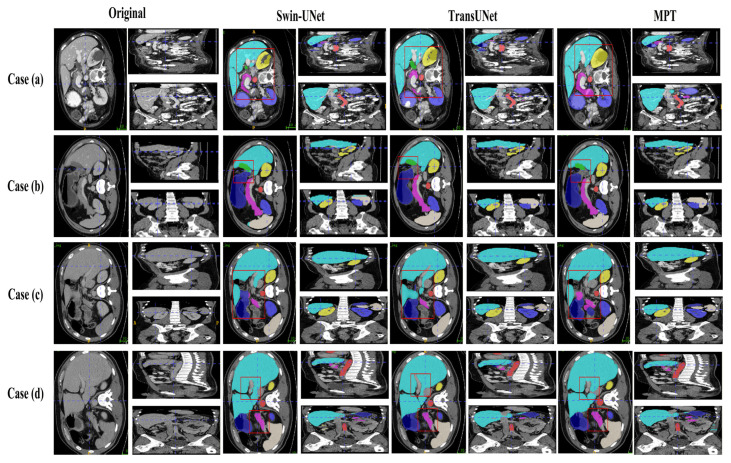
Visualizations of Synapse segmentation results from different angles.

**Figure 8:**
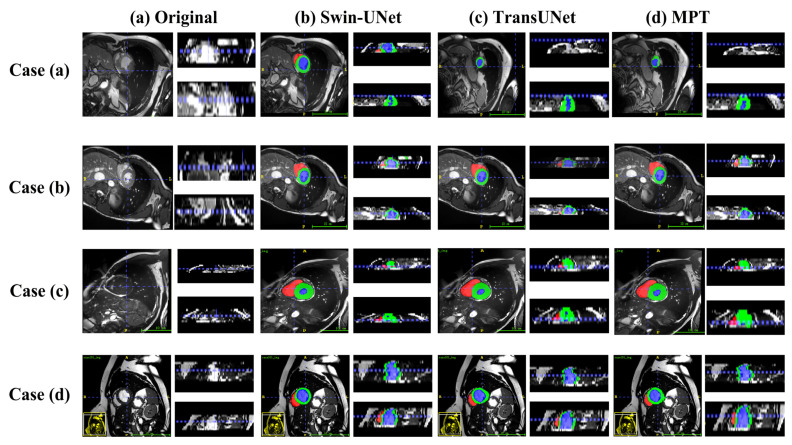
Visualizations of ACDC segmentation results from different angles.

Figure 7 and Figure 8 present qualitative comparisons of segmentation results on the Synapse and ACDC datasets.

On the Synapse dataset, the proposed MPT framework produces more accurate organ boundaries and cleaner regional predictions compared with traditional U-shaped CNN-based networks. In the updated visualization of Figure 7, the higher-resolution images make the liver–stomach interface and pancreas areas more distinguishable, allowing a clearer observation that MPT effectively suppresses false-positive predictions and achieves more precise boundary localization. Furthermore, when compared with transformer-based models such as TransUNet, MPT yields smoother and more continuous structural contours, reflecting its stronger global context encoding ability and improved semantic discrimination compared with existing methods.

On the ACDC dataset, as shown in the updated visualization of Figure 8, the proposed MPT model produces more regular ventricular shapes and clearer myocardial delineation compared with the baseline methods. The predicted myocardial ring appears more continuous and structurally coherent, and the boundaries of the ventricular cavities are smoother and more closely aligned with the ground truth. These improvements highlight the model’s enhanced ability to preserve anatomical structure and capture fine-grained cardiac contours.

These qualitative results confirm that MPT effectively integrates multi-scale contextual information and cross-dimensional feature refinement, enabling more precise segmentation while preserving detailed structural information across different imaging modalities.

## 4. Discussion

The medical image segmentation method proposed in this paper, based on a shift Multilayer Perceptron and Transformer, shows significant improvements in segmentation accuracy and efficiency compared to traditional methods like Unet. By preserving low-level features, introducing self-attention mechanisms, and enhancing skip connections, the proposed model not only achieves excellent results in single-organ segmentation but also demonstrates strong generalization capability in multi-organ segmentation, thereby greatly improving the efficiency of medical image analysis. The segmented medical images provide clearer insights into quantifying and evaluating organ lesion characteristics, offering doctors accurate tools for surgical planning. Furthermore, this method has made an important contribution to the development and deeper clinical application of medical imaging technology.

However, despite these promising results, several challenges and limitations remain. First, the non-uniformity, heterogeneity, and irregular shapes of tissues in medical imaging increase the difficulty of lesion segmentation. Second, with the rapid increase in medical data, issues such as computational complexity and memory usage when dealing with high-resolution images still pose significant challenges.

The performance of the proposed framework remains closely tied to the quality, scale, and diversity of the training data. When confronted with noisy, imbalanced, or limited datasets, its segmentation accuracy may decline due to insufficient feature representation. Although the current experimental outcomes demonstrate encouraging results, there is still considerable scope for improvement through further architectural refinement, parameter optimization, and task-oriented adaptation for specific medical imaging modalities.

In future work, we plan to extend the current 2D slice-wise design toward 2.5D and 3D variants to better capture inter-slice spatial continuity and volumetric contextual dependencies. Such extensions are expected to improve the model’s ability to represent fine anatomical structures across adjacent slices while maintaining computational efficiency through lightweight 3D attention or hybrid convolutional mechanisms. Integrating these volumetric enhancements with efficient training strategies will further expand the applicability of the framework to more complex medical imaging tasks.

Nonetheless, the proposed approach exhibits strong potential in complex and clinically relevant scenarios, particularly in situations where traditional segmentation algorithms tend to falter. Its ability to generalize across diverse organ structures highlights the model’s promise for real-world medical imaging applications. Future work will focus on enhancing data robustness, expanding dataset diversity, and adapting the framework to broader clinical contexts to fully unlock its potential.

## 5. Conclusions

This study proposes a novel Multilayer Perceptron–Transformer (MPT) framework for medical image segmentation that effectively balances global contextual reasoning and local structural preservation through the integration of Shift-MLP, Transformer encoding, and refined skip connections. The architecture enhances feature interaction and multi-scale representation via the SENet-based Atrous Spatial Pyramid Pooling (SASPP) and the Feature Aggregation Attention Module (FAAM). Extensive experiments on the Synapse (CT) and ACDC (MRI) datasets demonstrate that the proposed model achieves high-precision segmentation with lightweight computational efficiency (51.0 M parameters, 200 ms per slice). Statistical analyses further confirm the reliability of the improvements, and robustness tests under Gaussian noise and spacing perturbations show that Dice variations remain within 1.5%, indicating strong stability under real-world imaging variations.

In summary, the proposed MPT framework provides a robust, interpretable, and efficient solution for multi-organ medical image segmentation. It advances intelligent medical imaging analysis and offers practical value for computer-assisted diagnosis and treatment planning in clinical environments.

## Figures and Tables

**Table 1:** Samples selected in this work.

Dataset	Name	Number
Train	Synapse	2212 slices
	ACDC	1930 slices
Val	Synapse	1567 slices
	ACDC	551 slices

**Table 2:** Quantitative comparison of different networks on the Synapse dataset in terms of Dice Similarity Coefficient (DSC, %). The best performance is highlighted in bold, and the second best is underlined.

Methods	Aorta	Gallbladder	Kidney (L)	Kidney (R)	Liver	Pancreas	Spleen	Stomach
V-Net [41]	75.34	51.87	77.1	80.75	87.84	40.05	80.56	56.98
U-Net [16]	87.07	68.72	77.77	68.6	93.43	53.98	86.67	75.58
Swin-UNet [24]	85.47	66.53	83.28	79.61	94.29	56.58	**90.66**	76.6
Deeplabv3+ [42]	88.04	66.51	82.76	74.21	91.23	58.32	87.43	73.53
DARR [43]	74.74	53.77	72.31	73.24	94.08	54.18	89.90	45.96
TransUNet [9]	87.23	63.13	81.87	77.02	94.08	55.86	85.08	75.62
DSPNet [44]	**87.78**	62.22	78.01	74.54	69.32	57.76	69.31	72.06
DA-TransUNet [45]	86.54	65.2	81.70	80.45	94.5	**61.62**	88.53	79.73
VM-UNet [46]	86.40	**69.4**	**86.16**	**82.76**	94.1	58.80	89.51	**81.40**
CTC-Net [47]	86.46	63.53	83.71	80.79	93.7	59.73	86.87	72.39
**The proposed**	87.53	64.4	83.92	81.19	**94.8**	60.69	88.13	74.11

**Table 3:** Average performance of different networks across seven evaluation metrics (Dice Similarity Coefficient (DSC), 95% Hausdorff Distance (HD95), Recall, Precision, Sensitivity, Specificity, and Intersection over Union (IoU)).

Methods	V-Net [41]	U-Net [16]	Swin-UNet [24]	Deeplabv3+ [42]	DARR [43]	TransUNet [9]	The Proposed
DSC	68.81	69.77	79.13	77.63	69.77	77.48	**79.35**
HD95	36.5	39.7	21.55	39.95	23.4	31.69	**20.07**
Recall	64.87	73.46	77.72	91.22	86.24	76.64	**94.85**
Precision	79.29	82.83	93.20	91.02	95.87	85.39	**96.13**
Sensitivity	75.12	75.44	87.32	8641	**91.33**	77.01	89.63
Specificity	93.39	94.28	94.48	95.66	96.05	95.90	** 96.64 **
IOU	74.42	66.09	68.15	63.66	**81** **.** **13**	77.49	73.49

Bold formatting in the table indicates the optimal results. All values except HD95 are expressed as percentages (%); lower HD95 indicates better boundary accuracy. All values except HD95 are expressed as percentages (%); lower HD95 indicates better boundary accuracy.

**Table 4:** Quantitative comparison of different module combinations on the Synapse dataset in terms of Dice Similarity Coefficient (DSC, %).

Methods	Average	Aorta	Gallbladder	Kidney (L)	Kidney (R)	Liver	Pancreas	Spleen	Stomach
Baseline (TransUNet)	77.48	87.23	63.13	81.87	77.02	94.08	55.86	85.08	**75.62**
+Shift-MLP	75.60	85.73	62.07	80.88	74.03	93.34	53.13	83.52	72.10
+FAAM	77.14	87.33	63.95	78.30	73.63	93.83	61.15	87.70	70.86
+Spatial–Channel Skip	68.87	74.98	40.52	69.25	66.40	92.90	51.95	83.35	71.63
+SASPP	78.41	**87.92**	63.20	78.62	76.25	94.02	**64.16**	**89.94**	73.14
+CBAM (instead of FAAM)	76.23	83.44	60.12	75.79	74.98	91.33	61.74	88.71	70.49
+ASPP (instead of SASPP)	77.15	85.33	61.45	77.22	75.08	92.11	63.02	88.47	72.03
+FAAM + Skip	77.14	86.23	62.07	80.88	74.03	93.38	53.13	83.52	72.10
+SASPP + Skip	74.29	87.28	60.47	75.52	74.60	93.00	52.57	83.40	67.53
+SASPP + FAAM	75.90	85.73	62.06	80.87	72.12	93.21	57.79	83.32	72.10
Proposed MPT	**79.35**	87.53	**64.40**	**83.92**	**81.19**	**94.80**	60.69	88.13	74.11

Bold formatting in the table indicates the optimal results.

**Table 5:** Quantitative comparison of different module combinations on the ACDC dataset in terms of Dice Similarity Coefficient (DSC, %).

Methods	Average	RV	MYO	LV
Baseline	85.77	81.71	85.67	89.93
+Shift-MLP	85.85	81.5	85.51	90.55
+Shift-MLP + Skip	85.97	81.64	86.03	90.24
+Shift-MLP + Skip + FAAM	86.27	82.2	86.28	90.34
+Shift-MLP + Skip + SASPP	86.47	83.08	86.12	90.21
Proposed MPT	**87.01**	**83.32**	**86.83**	**90.90**

Bold formatting in the table indicates the optimal results.

**Table 6:** Independent *t*-test results of segmentation performance between baseline methods and the proposed MPT framework on Synapse and ACDC datasets.

Dataset	Compared Method	Baseline Mean ± SD	MPT (Ours) Mean ± SD	t	*p*	95% CI (Mean Diff.)
Synapse	V-Net	76.8 ± 0.7	78.8 ± 0.5	3.01	0.016	[+0.42, +3.68]
	DARR	77.7 ± 0.8	78.8 ± 0.5	2.02	0.078	[−0.18, +2.18]
	TransUNet	78.2 ± 0.6	78.8 ± 0.5	1.46	0.183	[−0.33, +1.47]
	DSPNet	78.1 ± 0.8	78.8 ± 0.5	1.38	0.205	[−0.38, +1.68]
ACDC	U-Net	84.6 ± 0.8	86.5 ± 0.6	2.87	0.020	[+0.32, +3.28]
	Attention-UNet	85.4 ± 0.7	86.5 ± 0.6	2.06	0.073	[−0.15, +2.25]
	Swin-UNet	86.1 ± 0.6	86.5 ± 0.6	1.22	0.256	[−0.44, +1.24]
	TransUNet	85.9 ± 0.7	86.5 ± 0.6	1.46	0.181	[−0.34, +1.54]

**Table 7:** Efficiency comparison of different segmentation frameworks on the Synapse dataset.

Model	Params (M)	FLOPs (G) (per 512 × 512 Slice)	Inference Time (ms/Slice) (FP32/AMP)	Peak VRAM (GB) (FP32/AMP)
TransUNet	105.2	≈680	290/230	7.4/4.6
Swin-UNet	62.1	≈500	230/185	6.3/4.1
MPT (ours)	51.0	≈325	200/160	5.2/3.6

**Table 8:** Quantitative robustness analysis of the proposed MPT framework under simulated Gaussian noise and voxel spacing perturbations on the Synapse dataset.

Perturbation Type	Perturbation Level	Dice (%)	HD95 (mm)
Baseline	–	79.35	20.07
Gaussian Noise	σ = 0.01	78.87	20.56
Gaussian Noise	σ = 0.03	78.05	21.22
Gaussian Noise	σ = 0.05	77.01	22.41
Spacing Perturbation	±5%	78.63	20.84
Spacing Perturbation	±10%	78.02	21.36

## Data Availability

The data presented in this study are available on request from the corresponding author.

## Abbreviations

The following abbreviations are used in this manuscript:

MPT	Multilayer Perceptron Transformer
SASPP	Senet Atrous Spatial Pyramid Pooling
FAAM	Feature Aggregation Attention Module
DWConv	Depthwise Convolution
DSC	Dice Similarity Coefficient
HD95	95% Hausdorff Distance
ACDC	Automatic Cardiac Diagnostic Challenge (Dataset)
CE	Cross Entropy
ASPP	Atrous Spatial Pyramid Pooling
DARR	Domain Adaptive Relational Reasoning Network
CBAM	Convolutional Block Attention Module
DSPNet	Dual-Scale Perception Network
